# ChatMol: interactive molecular discovery with natural language

**DOI:** 10.1093/bioinformatics/btae534

**Published:** 2024-09-02

**Authors:** Zheni Zeng, Bangchen Yin, Shipeng Wang, Jiarui Liu, Cheng Yang, Haishen Yao, Xingzhi Sun, Maosong Sun, Guotong Xie, Zhiyuan Liu

**Affiliations:** Department of Computer Science and Technology, Tsinghua University, Beijing 100084, China; Department of Computer Science and Technology, Tsinghua University, Beijing 100084, China; PingAn Technology, Beijing 100027, China; PingAn Technology, Beijing 100027, China; School of Computer Science, Beijing University of Posts and Telecommunications, Beijing 100876, China; PingAn Technology, Beijing 100027, China; PingAn Technology, Beijing 100027, China; Department of Computer Science and Technology, Tsinghua University, Beijing 100084, China; PingAn Technology, Beijing 100027, China; Department of Computer Science and Technology, Tsinghua University, Beijing 100084, China

## Abstract

**Motivation:**

Natural language is poised to become a key medium for human–machine interactions in the era of large language models. In the field of biochemistry, tasks such as property prediction and molecule mining are critically important yet technically challenging. Bridging molecular expressions in natural language and chemical language can significantly enhance the interpretability and ease of these tasks. Moreover, it can integrate chemical knowledge from various sources, leading to a deeper understanding of molecules.

**Results:**

Recognizing these advantages, we introduce the concept of conversational molecular design, a novel task that utilizes natural language to describe and edit target molecules. To better accomplish this task, we develop ChatMol, a knowledgeable and versatile generative pretrained model. This model is enhanced by incorporating experimental property information, molecular spatial knowledge, and the associations between natural and chemical languages. Several typical solutions including large language models (e.g. ChatGPT) are evaluated, proving the challenge of conversational molecular design and the effectiveness of our knowledge enhancement approach. Case observations and analysis offer insights and directions for further exploration of natural-language interaction in molecular discovery.

**Availability and implementation:**

Codes and data are provided in https://github.com/Ellenzzn/ChatMol/tree/main.

## Introduction 

Molecular design is a crucial task in fields such as biochemistry and material science. This area has seen significant advancements in recent years due to the development of deep learning technologies (Wang *et al.* 2022). Typically, existing systems either generate new molecules or optimize the given ones (Du et al. 2022) using various chemical representations, such as the Simplified Molecular Input Line Entry System (SMILES; Weininger 1988) and structural formulas. However, these chemical languages often lack readability and require extensive expertise, posing a challenge for users. Additionally, current molecular design systems lack interactivity and fail to integrate functions such as retrieval and editing effectively, making the process both difficult and time-consuming, despite the assistance of deep learning methods.

The emergence of pretrained language models (PLMs; Han *et al.* 2021), and especially large language models (LLMs) (https://platform.openai.com/docs/model-index-for-researchers), introduces new possibilities for this field. These models demonstrate substantial capabilities in delivering extensive biochemical knowledge and responding to complex human instructions (Tong and Zhang 2023). This suggests that natural language processing (NLP) systems could be leveraged to communicate molecular design requirements more effectively. Additionally, integrating factual information from external natural language sources (e.g. biochemical literature) is crucial as it provides complementary insights into the molecular structures and mechanisms described by chemical languages.

Current research has begun to explore the application of NLP methods to molecular tasks, including self-supervised architectures for molecule comprehension (Goh *et al.* 2018; Rong *et al.* 2020; Wang *et al.* 2019). As for using natural language in molecular design, several tasks have been proposed, such as molecule-description matching (Zeng *et al.* 2022), molecule captioning, and generation from descriptions (Edwards *et al.* 2022). Nevertheless, these tasks vary significantly and lack the flexibility and scalability needed to fully utilize natural language as an interactive medium for molecular manipulations.

**Figure 1.**
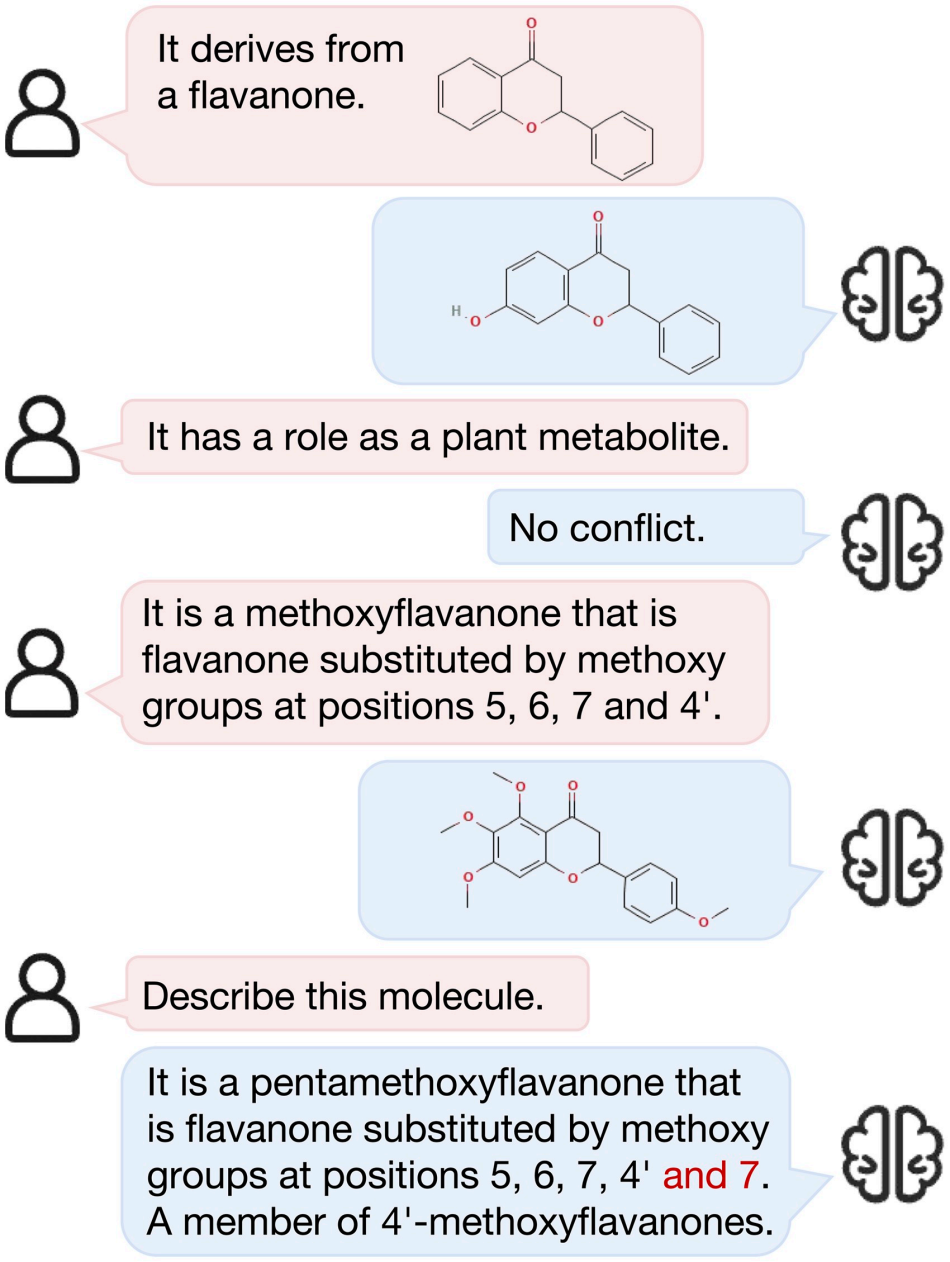
Conversational molecular design diagram.

We propose a new approach, termed conversational molecular design, which allows users to interact freely, providing molecular information in either chemical or natural language, as shown in Fig. 1. This approach facilitates the generation of readable property descriptions or the modification of molecules to meet specific requirements through multi-turn conversations. To support the training and evaluation of conversational molecular design, we create a dataset for conversational molecular design, based on the molecule-description parallel data and manually designed rule filters.

Although PLMs have shown their capability to process complicated natural-language commands, conversational molecular design remains a challenging task. Specifically, while PLMs can effectively handle natural and chemical languages when used separately, conversational design demands a flexible and synergistic understanding of both types of text. Moreover, given the specialized nature of chemical materials, relying solely on commonsense knowledge is insufficient for molecular design tasks.

To address these challenges, we introduce ChatMol, a model based on a generative PLM that can deeply understand and generate natural language (Raffel *et al.* 2020). ChatMol is specifically enhanced to smoothly bridge natural and chemical languages, facilitating interactive conversations, and to integrate extensive molecular knowledge for more effective molecule design.

For bridging different languages, we train ChatMol to concurrently process natural and chemical languages, identifying intricate associations between them with minimal supervision. Initially, we treat SMILES strings as regular text and conduct masked language modeling on both SMILES and biochemical literature to develop a fundamental understanding of each language. Additionally, ChatMol is trained to recognize chemical entities in natural language texts and convert these entities into their respective chemical language expressions. To automatically generate training data, we utilize chemical named entity recognition tools to detect entities in literature and retrieve their corresponding SMILES strings from existing databases.

For injecting molecular knowledge, we incorporate two types of sophisticated yet representative knowledge into ChatMol to better handle specialized molecular tasks. This includes *experimental properties* from knowledge bases (KBs), such as physical and chemical properties derived from wet-lab experiments, and *spatial structures* calculated using toolkits. The latter involves understanding chemical bonds, molecular rings, and aromaticity, which enriches the representation of spatial structure information in SMILES expressions. Given the chemical SMILES, our model is trained to generate natural language answers constructed with the detailed molecular knowledge.

Our main contributions are as follows: (1) innovative task proposal: We introduce conversational molecular design, a novel task that leverages natural language as the primary medium for human–machine interaction, facilitating accessible molecular discovery; (2) model development: We present ChatMol, a pretrained model specifically enhanced to excel in this new task through the integration of molecular knowledge and associations between chemical and natural languages; (3) empirical validation: Experimental results not only highlight the complexity of the task we propose but also demonstrate the efficacy of our approach, achieving superior performance with significantly fewer pretraining steps than traditional methods.

## Related work

### Knowledgeable models

Though the deep learning models have the ability to learn commonsense knowledge from large-scale training data in an unsupervised manner, it is more direct to enhance the model by incorporating knowledge to models. Specific to the biochemical domain, researchers replace the pre-training corpus with biochemical data to transfer the general PLMs to this domain (Wang *et al.* 2023a), while knowledgeable training and inferencing methods are still proven to be useful. There are two mainstream knowledgeable approaches: (1) Explicitly encode the knowledge to assist the models. Extra modules are usually targeting designed for the downstream task. For instance, related tags can be acquired from knowledge graphs (KGs) and separately encoded to get knowledge fusion attention for the chemical–protein interaction extraction (Sun *et al.* 2020). KGs information can also be incorporated via a hierarchical graph representation to modify the PLM contextualized embeddings for biomedical entity/relation/event extraction (Huang *et al.* 2020; Lai *et al.* 2021). (2) Implicitly teach the knowledge with special pretraining tasks. Knowledge items can help prepare data for multi-task training with methods such as distant supervision. Based on KG items, a binary classification task is added to predict whether a triplet exists or not (Hao *et al.* 2020), and entity detection and linking tasks are conducted for similar purposes (Yuan *et al.* 2021).

When it comes to SMILES strings processing, since SMILES cannot fully represent the topological information of molecules, supplementary knowledge enhancement, especially spatial knowledge, is emphasized (Zhang *et al.* 2021). Graph representations (Elton *et al.* 2019) are popular with molecular deep learning research, because they do not have to consider rotation, permutation, and other issues additionally compared with other methods such as atom coordinates. Some graph-based methods also incorporate 3D spatial information (Danel *et al.* 2020), using the universal force field to generate the conformations. Geometry-enhanced molecular representation learning (Fang *et al.* 2022) explores the effect of using different force field constraints (e.g. Merck molecular force field (Halgren 1996) and Density functional theory (Parr 1983)) on the quality of the generated coordinates.

### Models bridging versatile subdomains

For bridging different models, there exist countless methods, especially in the image-text field. Pretrained models such as ontrastive Language–Image Pre-training (Radford *et al.* 2021) and Unimo (Li *et al.* 2021) have brought the fusion of the two models to a new level. With similar ideas, we can also bridge data from various subdomains that have natural gaps. To process different forms of biochemical data, one solution is to convert them into the same modal and form. For example, Goh *et al.* (2018) unified the molecular graph data and SMILES data to molecular structure images as the model input; KV-PLM (Zeng *et al.* 2022) is the first research trying to fuse SMILES strings and natural language descriptions together to improve the knowledge reserve and chemical comprehension of the model; MolT5 (Edwards *et al.* 2022) further develops the idea to conduct translation between molecules and text descriptions by a generative model; Su *et al.* (2022) proposed a cross-modal graph-based model linking molecular and natural language, while it ignores the flexibility of natural language descriptions. Overall, bridging chemical and natural language is an innovative scenario that has drawn attentions but not been fully explored.

### LLMs for chemical tasks

In the past 2 years, general-domain LLMs are adopted to molecular tasks with the professional knowledge injected into their prompts (Li *et al.* 2024a). Domain-specific LLMs such as ChemDFM (Zhao *et al.* 2024) have been developed for disciplines like chemistry (Zhang *et al.* 2024). These models are primarily constructed by utilizing resources like chemical literature and databases, along with the automatically annotated prompts (Guo *et al.* 2023) from general LLMs (e.g. ChatGPT), to automatically generate vast amounts of instruction-tuning data. This approach allows the models to retain generalizability while acquiring specialized chemical capabilities, and they are capable of performing tasks like formula transformations and specific property prediction. Additionally, following our preprint work, there have been efforts on interactive molecular editing, such as DrugAssist (Ye *et al.* 2023). This tool leverages the LLaMA-2-7B-chat model (Touvron *et al.* 2023) and learns KB information to accurately modify molecules based on properties like solubility. Models including GIT-Mol (Liu *et al.* 2024) and 3D-MOLM (Li *et al.* 2024b) take a step in integrating materials from data forms apart from natural language, such as 3D molecular structures and images. These subsequent works underscore the pioneering nature of interactive molecular design and demonstrate the utility of knowledge-enriched training in enhancing the applicability of larger foundational models.

## System and methods

**Figure 2.**
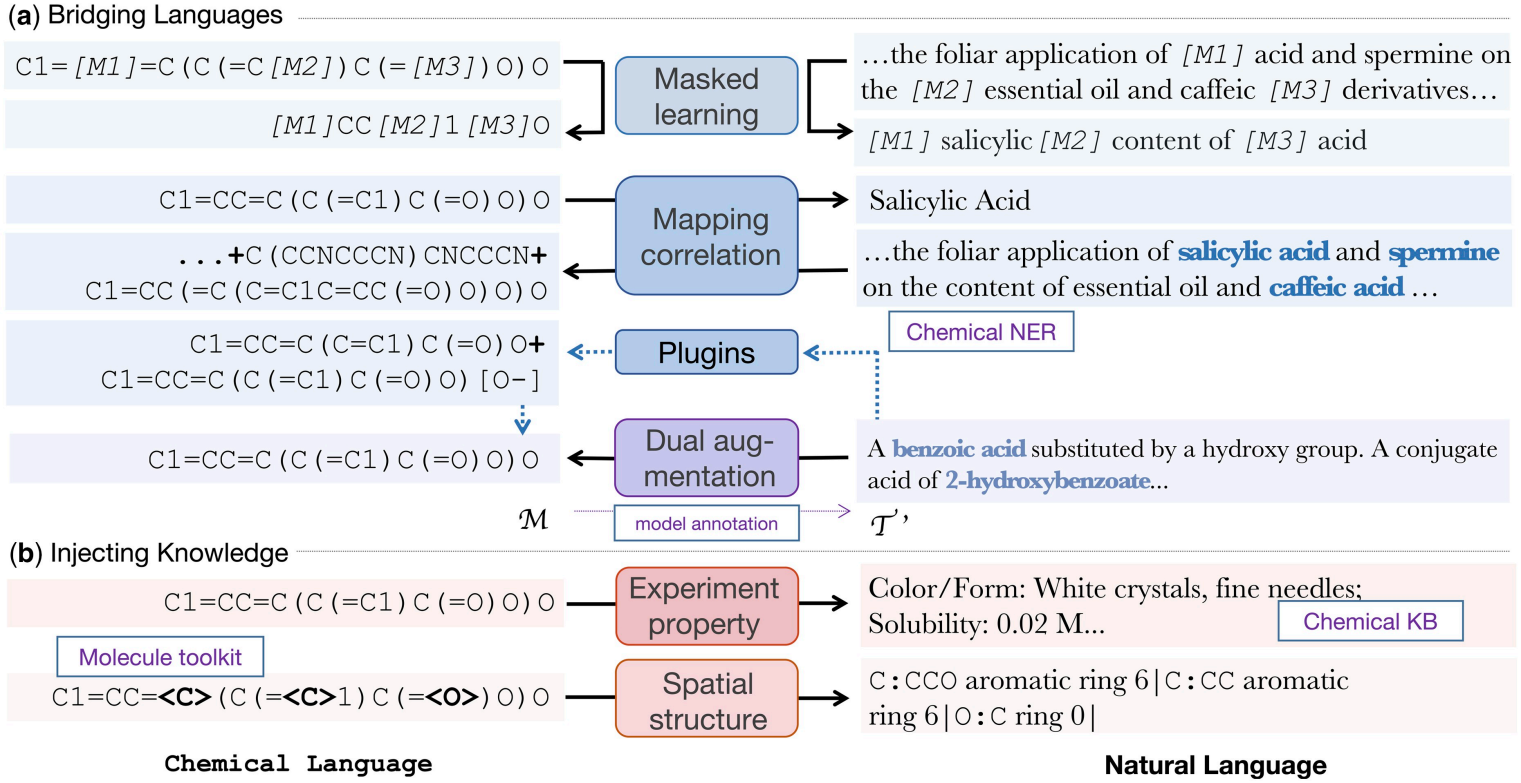
Knowledgeable and versatile training process for ChatMol. The black arrows indicate the input to output direction of the task.

In this section, we clarify the definition and related settings of the newly proposed task. Then, we present the knowledgeable and versatile training method of ChatMol which is designed to better finish the conversational molecular design task. The overall process is shown in Fig. 2. We mix up all the data with different task prefixes in the period of multi-task pretraining and apply the plugins mentioned in molecule mapping correlation in the period of fine-tuning.

### Task definition and evaluation metrics

In each turn of the molecular design, both the human user and the intelligent system may refer to molecules by chemical language, or to chemical properties by natural language. Given the conversation history H(M,T) which contains molecules $M_{1,2,\ldots ,p}$ and chemical property descriptions $T_{1,2,\ldots ,q}$, we focus on two main functions that researchers expect the intelligent system to do:

(1) Molecule understanding: The system is required to generate a paragraph of property descriptions $T_{i}^{\prime }$ for the molecule $M_{i}$ in *H*. (2) Molecule generation: The system is also supposed to generate a specific molecule ${M^{\prime }}_{j}$ that meets the requirements in *H*, which may be described by natural language or by molecules that the target has to be similar with. Since there may be more than one molecule matching the descriptions, the human user can iteratively replenish property descriptions $T_{j+1}$, and the system would generate a modified molecule ${M^{\prime }}_{j+1}$ according to the current text descriptions $T_{1,2,\ldots ,j+1}$ and the last-round result ${M^{\prime }}_{j}$ (with the input as ‘it looks like ${M^{\prime }}_{j}$).

For traditional text generation tasks, evaluation metrics such as BLEU (Papineni *et al.* 2002), ROUGE (Lin 2004), and METEOR (Banerjee and Lavie 2005) are widely used to measure the similarity of the predicted results and the reference answers. BLEU scores are mainly used to evaluate the precision of generated content; ROUGE scores mainly evaluate the completeness (recall) of the content; METEOR scores take into account synonyms through external knowledge sources and mainly evaluate the meaning similarity of the content. They can also be applied to the evaluation of molecule understanding. However, for the molecule generation task, the content textual similarity is not persuasive from the perspective of the accuracy of molecules.

For molecule generation, in addition to using the BLEU score to measure the text precision, we mainly consider two aspects: Matching rate and molecular similarity. For the matching rate, we ask the model to generate the three most likely molecules for each input and calculate the frequency of hits for the first one molecule (exact match accuracy) and the first three molecules (hit@3 accuracy). For the molecular similarity, we apply various fingerprint systems for evaluation, such as various fingerprint Tanimoto similarity (FTS) metrics (Tanimoto 1958; Schneider *et al.* 2015; Durant *et al.* 2002). They detect similar functional substructures in the molecules and can indicate the similarity of properties.

### Model and data preparation

Since the features of SMILES and natural language are quite different, we prepare two sets of encoder and decoder to separately process M and T. Each set is set as a common sequence-to-sequence (seq2seq) framework and initialized with T5-base (Raffel *et al.* 2020), a widely adopted seq2seq PLM with 220M parameters.

As for the data preparation, the corresponding 〈M,T〉 pairs can be acquired from the chemical KBs, in which brief text introductions of substances are provided. Information leakage may happen due to the appearance of molecule names in the text. To alleviate the problem, synonyms of the target molecule are replaced by general referential phrases such as ‘the molecule’.

To conduct the multi-turn molecule generation, we create a new dataset ChEBI-dia based on ChEBI-20. In the original dataset, T used to describe the molecule, M usually contains multiple sentences, in the order of description from fine structures to overall properties. To get the multi-turn text descriptions, we split T into sentences and reverse their order to get $S_{1,2,\ldots ,n}$ in which the amount of detailed information increases sequentially. For the *k*th turn, we have $T_{k}=\{S_{1},S_{2},\ldots ,S_{k}\}$. To get the molecule intermediate results, we adopt the pretrained MolT5-caption2smiles-large model (Edwards *et al.* 2022) to automatically generate five candidates $M_{k1,k2,\ldots ,k5}$ for the given $T_{k}$, and randomly pick one as the molecule $M_{k}$ expected to be generated, which has RDK fingerprint similarity (Landrum *et al.* 2013) with the final answer $M_{n}$ higher than 0.5 while lower than 1 (i.e. to avoid information leakage).

Additionally, we filter out those items that contain only one turn of conversation, and delete those with ‘-’ in the sentences to avoid the appearance of the standard chemical nomenclature (e.g. IUPAC nomenclature) to directly reveal the answer. Besides, we randomly remain a few items that the intermediate molecules have low similarities with the final answers to keep the variety and help enhance the model’s robustness.

In order to verify the representativeness of the dataset, we analyzed the similarity between all the molecules involved. The average similarities of RDK molecular fingerprints within and between training and test data are all <0.18; <32% of the test molecules have appeared in the training set, and there are a few macromolecules with a molecular weight of more than 5000 g/mol. Therefore, the molecules involved cover a wide range and the test scenarios are sufficiently generalized (for more details such as the source, data volume, and specific composition of the pretraining corpus, refer to Supplementary Data).

### Bridging different languages

We first conduct the popular pretraining paradigm masked language modeling (MLM) to ensure the basic comprehension and generation capability of our model isolated on natural and chemical languages. However, to process the multi-modal conversational molecular design data, it is also necessary for our model to capture the associations and convert between the two languages. Thus, we apply the following two strategies to construct parallel data for training.

#### Molecule mapping correlation

We detect the molecular entities in the literature corpus with the SciSpacy tool (Neumann *et al.* 2019) and then retrieve for their SMILES from PubChem (Kim *et al.* 2016). Given the natural language segments, the molecule generation model is required to generate SMILES expressions for all the molecules that appeared in it sequentially. Conversely, the molecule understanding model is expected to generate the standard names of the given molecules. In this way, we help ChatMol build the parallel association with the least supervision.

Notice that the toolkit and KB can also play a role as plugins during the downstream fine-tuning and inferencing. For the text descriptions given for molecule generation, we provide the automatically annotated entity SMILES strings as the model prompts. To avoid information leakage, we forcefully remove those SMILES strings in the prompts that are the same as molecules in the answers.

#### Dual augmentation

Considering that the molecule understanding (molecule-to-text generation) and molecule generation (text-to-molecule generation) are a pair of mutual tasks, while existing molecule SMILES strings are much more than molecular property descriptions in natural language, we can take the idea of dual learning (He et al. 2016; Xia *et al.* 2017), a common mechanism in neural machine translation, to alleviate the challenge of lacking parallel data. Specifically, the fine-tuned molecule understanding model can generate augmented text descriptions for any given molecule, which can feedback on the molecule generation training.

### Injecting molecular knowledge

To get an in-depth comprehension of the given chemical language expressions and to generate more reasonable and informative natural language descriptions, our model is trained with two types of molecular knowledge. Given the molecule SMILES, our model is required to generate the corresponding knowledge as shown in Fig. 2.

#### Experimental property knowledge

We expect the model to understand the molecular structures deeply to infer the molecular properties that may be explicitly described in the natural language. To do so, we collect 15 types of physical and chemical properties determined experimentally from PubChem database (Kim *et al.* 2016), including solubility, color, corrosivity, and so on. These properties can be directly described in natural language and provide supervision signals for molecule understanding.

#### Spatial structure knowledge

Spatial information about molecules is essential for understanding molecular properties, while SMILES expressions do not directly express the topological structure of molecules in the language model. To cope with the demand of understanding molecule structures, we introduce spatial-related pre-training tasks. We use the RDKit toolkit (Landrum *et al.* 2013) to get the spatial structure of the input molecule. For the specific atom, the model is required to recognize the other atoms connected to it, as well as its aromaticity and ring formation information.

## Results and discussion

In this section, we first introduce the basic settings including datasets and baseline models. Next, we introduce the molecule understanding and generation experiments and the corresponding ablation study. To evaluate the model capability more comprehensively, we also provide the case analysis.

### Basic settings

For molecule understanding, there are few datasets focusing on mutual generation between text and molecule, such as PCdes (Zeng *et al.* 2022) and ChEBI-20 (Edwards *et al.* 2022). We adopt both as our fine-tuning and evaluation datasets. There are 26 407 train, 3301 validation, and 3300 test pieces of molecule-description pairs in ChEBI-20. Each piece of description has 43.4 words and 3.3 sentences on average. Correspondingly, there are 10 500 train, 1500 validation, and 3000 test pieces in PCdes. Descriptions in this dataset are longer than in ChEBI-20, with 62.1 words and 4.3 sentences on average.

For molecule generation, we evaluate on our newly proposed ChEBI-dia conversation dataset. We have 7361 multi-turn dialogs for training, 1369 for validation, and 1311 for test. There are altogether 7626 2-turn dialogs, 4536 3-turn dialogs, 1363 4-turn dialogs, 283 5-turn dialogs, and 20 even longer dialogs. The sentence characteristics are not much different from the original ChEBI-20 dataset.

We evaluate the following models:

*ChatGPT*: This refers to the ‘gpt-3.5-turbo’ model in the OpenAI series and is one of the representative LLMs. For molecule understanding, we provide three randomly picked examples to tell the exact language style of the specific datasets in the prompt. For molecule generation, we only provide one instance to constrain the output format of the model.

*KV-PLM*: We adopt the model checkpoint provided in the original paper (Zeng *et al.* 2022), which is a versatile model bridging natural and chemical languages while based on SciBERT (Beltagy *et al.* 2019), a popular PLM in the biomedical domain. Since KV-PLM is an encoder model, we manage to choose alternative retrieval solutions to compare the capabilities of the generation task as fairly as possible. The model is trained with contrastive learning matching molecules and descriptions. It retrieves the best-matched molecules among train, validation, and test sets for molecule generation, and retrieves sentences from the train set to compose the description paragraphs for molecule understanding.

*T5*: This is the backbone of our model. We directly fine-tune T5-base on the downstream datasets.

*MolT5*: We adopt the model checkpoint provided in the original paper (Edwards *et al.* 2022) which is initialized with T5-base checkpoint and then pre-trained by MLM on molecules and texts with 1 million steps (batch size as 256).

*ChatMol(ours)*: We initialize ChatMol with T5-base and conduct multi-task pre-training. For the molecule understanding version, we adopt tasks including MLM, mapping correlation, experimental property and spatial structure, and pretrain altogether 6000 steps (batch size as 256). For the molecule generation version, we adopt MLM and mapping correlation learning tasks for also altogether 6000 steps. Notice that the molecule generation version is first tuned on the augmented data produced by the opposite model, and then fine-tuned on ChEBI-dia.

*ChatMol+(ours)*: This is the same model as above, while we allow the plugin use of detecting entities and retrieving their SMILES with toolkits and KBs in the molecule generation task, as introduced in Section 3. The prompted SMILES can provide mapping knowledge between natural and chemical languages in a targeted manner.

Details for training settings are introduced in Supplementary Data.

### Result analysis

Results for the main experiment are shown in Tables 1 and 2. Our model has achieved all the best performances under different settings and metrics. Details for the intermediate-turn results of molecule generation are introduced in Supplementary Data.

**Table 1.** Molecule understanding experiment results. The arrow indicates the direction in which the metric is better. The bold values show the best results.

Model	Training steps	Dataset	BL-2↑	BL-4↑	RG-1↑	RG-2↑	RG-L↑	MET↑
ChatGPT		ChEBI	0.226	0.121	0.311	0.117	0.232	0.292
		PCdes	0.144	0.055	0.244	0.062	0.158	0.233
KV-PLM	0	ChEBI	0.406	0.319	0.506	0.323	0.430	0.506
		PCdes	0.235	0.145	0.331	0.143	0.250	0.323
T5	0	ChEBI	0.630 ± 0.001	0.553 ± 0.002	0.665 ± 0.003	0.520 ± 0.005	0.604 ± 0.003	0.632 ± 0.002
		PCdes	0.374 ± 0.014	0.289 ± 0.014	0.440 ± 0.003	0.275 ± 0.005	0.377 ± 0.003	0.393 ± 0.010
MolT5	1 million	ChEBI	0.626 ± 0.026	0.549 ± 0.030	0.661 ± 0.021	0.517 ± 0.026	0.601 ± 0.021	0.628 ± 0.023
		PCdes	0.343 ± 0.043	0.262 ± 0.042	0.433 ± 0.033	0.264 ± 0.032	0.368 ± 0.030	0.369 ± 0.038
**ChatMol**	6000	ChEBI	**0.647** ± 0.002	**0.573** ± 0.004	**0.678** ± 0.001	**0.538** ± 0.002	**0.618** ± 0.001	**0.649** ± 0.003
		PCdes	**0.396** ± 0.021	**0.312** ± 0.018	**0.458** ± 0.005	**0.295** ± 0.003	**0.395** ± 0.004	**0.414** ± 0.012

*Note*: BL, BLEU; RG, ROUGE; MET, METEOR. We display the average results under five different random seeds. ChatMol demonstrated a statistically significant improvement over the T5 and MolT5 baseline, with *P* < .05 and Cohenś *d* > 1.5.

**Table 2.** Molecule generation experiment results. The arrow indicates the direction in which the metric is better. The bold values show the best results.

Model	Training steps	EM↑	hit@3↑	BL↑	Leven↓	RDK↑	MAC↑	Morgan↑
ChatGPT		0.009	0.009	0.405	56.27	0.283	0.483	0.184
KV-PLM	0	0.111	0.223	0.654	34.14	0.532	0.691	0.418
T5	0	0.052 ± 0.007	0.078 ± 0.012	0.600 ± 0.032	40.71 ± 0.802	0.513 ± 0.085	0.672 ± 0.036	0.419 ± 0.046
MolT5	1 million	0.061 ± 0.011	0.085 ± 0.019	0.605 ± 0.022	40.47 ± 0.810	0.517 ± 0.040	0.667 ± 0.032	0.422 ± 0.032
**ChatMol**	6,000	0.084 ± 0.010	0.117 ± 0.017	0.626 ± 0.033	38.35 ± 0.505	0.579 ± 0.050	0.716 ± 0.052	0.485 ± 0.040
**ChatMol+**	6,000	**0.140** ± 0.008	**0.183** ± 0.013	**0.712** ± 0.075	**29.89** ± 0.646	**0.649** ± 0.079	**0.764** ± 0.041	**0.551** ± 0.051

*Note*: EM, Exact Match. Leven, Levenshtein distance. RDK, RDK Fingerprint Similarity. MAC, MACCS Fingerprint Similarity. Morgan, Morgan Fingerprint Similarity.

Average results under five different random seeds are displayed. ChatMol demonstrated a statistically significant improvement over the T5 and MolT5 baseline, with *P* < .05. ChatMol+ demonstrated a statistically significant improvement over the ChatMol, T5, and MolT5 baseline, with *P* < .05 and Cohenś *d* > 1.5.

#### Molecule understanding results

For molecule understanding, the T5-based generative models consistently outperform other model types, achieving notably better performance, and our model further achieves a slight improvement. As shown in Table 3, the LLM baseline ChatGPT, while proficient in natural language generation and possessing a substantial grasp of chemical knowledge, frequently commits errors on relatively straightforward facts (e.g. incorrectly stating that iodoacetic acid is a liquid). This underscores the challenges inherent in this task, as the evaluation scores are not on par with those of the fine-tuned models. The encoder model, KV-PLM, also fails to show any advantage in generating accurate descriptions, potentially due to the limitations imposed by its encoder architecture on the diversity of descriptions it can produce. In contrast, models such as T5, MolT5, and ChatMol, all consistently generate reasonable property descriptions for the given molecules. Despite statistical analyses confirming significant improvements through our method, visual inspections often do not show marked differences in many instances. This suggests that the model architecture and the choice of fine-tuning data are more crucial for performance in molecule understanding tasks than the specifics of the pretraining approach. It is important to note that MolT5 shows reduced performance on PCdes compared with ChEBI-20 provided by the original paper, which may suggest that excessive pretraining could detrimentally affect the model’s generalization abilities. In addition, MolT5 encountered checkpoints with poor performance when randomly repeated training on the two datasets. Its performance was not stable, which may be related to the training method.

**Table 3.** Examples of ChatGPT applying to conversational molecular design.

Molecule understanding
<INPUT> You are now a chemical assistant. I will provide you with a mole-cule SMILES string, and you have to return a paragraph of proterty descriptions for this molecule. For example:
Molecule: CCCC[Si](Cl)(Cl)Cl
Description: It appears as a colorless liquid with a pungent odor. Flash point 126Â°F. Corrosive to metals and skin. Used to make various silicon contain-ing compounds…
Now the Molecule is: C(C(=O)O)I. What is the Description?
<OUTPUT> The molecule is an organic compound that belongs to the class of carboxylic acid derivatives. Appears as a colorless to pale yellow liquid with a pungent odor and can be synthesized by treating iodine with acetic anhydride. It is known to be corrosive to metals and can cause severe irritation…

Molecule Generation

<INPUT> You are now a chemical assistant. I will provide you with a paragraph of property descriptions, and you have to return a SMILES string of a molecule that meet the requirements. For example:
Description: It is an acetate ester, a cyclic terpene ketone, a member of furans, a limonoid and a tetracyclic triterpenoid.
Molecule: CC(=O)O[C@@H]1C[C@@H]2[C@](C=CC(=O)C2(C)C)([C@@H] 3[C@@]1(C4 = CC[C@H]([C@@]4(CC3)C)[C@@H]5CC(=O)OC5)C)C
Now the Description is: It has a role as a food acidity regulator and a fundamental metabolite. It is an intermediate metabolite in the citric acid cycle. It looks like C(C(C(=O)O)C(=O)O)C(=O)O. What is the Molecule?
<OUTPUT> The molecule is: C(CC(=O)O)C(C(=O)O)C(=O)O.

#### Molecule generation results

For molecule generation, our method significantly outperforms the baseline models in generating results that closely match the ground truth molecules. As shown in Table 3, the LLM baseline ChatGPT demonstrates relatively low hit scores and fingerprint similarities, indicating its limited capability to generate molecules that are identical or similar to the desired answers. We know from observation that ChatGPT can produce valid molecules and possesses some molecular knowledge, while the scope of its capabilities is somewhat restricted, and the range of predictions is narrow. KV-PLM, in contrast, employs a retrieval-based approach, selecting the best-matched molecules from predefined pools, which allows it to achieve higher hit scores. However, this method is less effective in open-ended scenarios where such predefined pools may not be available. Among the T5-based models, MolT5 shows only marginal improvement over the original T5 model. Meanwhile, ChatMol, particularly the version enhanced with SMILES strings obtained through plugins, marks a significant leap in performance. The Exact Match score for ChatMol is nearly triple that of the original T5, effectively reducing the time users spend to identify their target molecules by a factor of three. These results underscore the critical role of bridging different languages and the efficiency of utilizing pretrained models augmented with reliable tools in enhancing the accuracy and applicability of molecule generation.

#### Ablation study

We remove our knowledgeable strategies sequentially to show their effectiveness. As shown in Table 4, *w/o property*, *w/o spatial*, and *w/o mapping* refer to the removal of experimental property prediction, spatial structure prediction, and molecule mapping correlation in the multi-task pretraining; *w/o conversation* refers to the removal of molecules in the conversation history; *w/o augmentation* refers to removing the dual augmentation training; *w/o prompting* refers to removing the SMILES prompts obtained from plugins.

**Table 4.** Ablation study results. The bold values show the best results.

Understanding	BL-2	BL-4	RG-L
Ours	**0.647**	**0.573**	**0.618**
*w/o property*	0.641	0.565	0.612
*w/o spatial*	0.640	0.565	0.613
*w/o mapping*	0.632	0.556	0.607
*w/o all*	0.630	0.553	0.604

Generation	EM	BL	RDK

Ours	**0.140**	**0.712**	0.649
*w/o conversation*	0.129	0.710	0.640
*w/o augmentation*	0.136	0.696	**0.650**
*w/o mapping*	0.130	0.681	0.633
*w/o prompting*	0.084	0.626	0.579
*w/o all*	0.052	0.600	0.513

As we can see, all the non-full versions of ChatMol perform worse on both tasks, proving the effectiveness of our method. Especially, the mapping correlation training and the SMILES prompting show significantly lower scores when being removed, showing that the capability of bridging versatile subdomains is essential to accomplish the conversational molecular design. The *w/o conversation* version proves that the iterative modification form is more reasonable than directly providing a whole paragraph of text requirements.

### Case study

**Figure 3.**
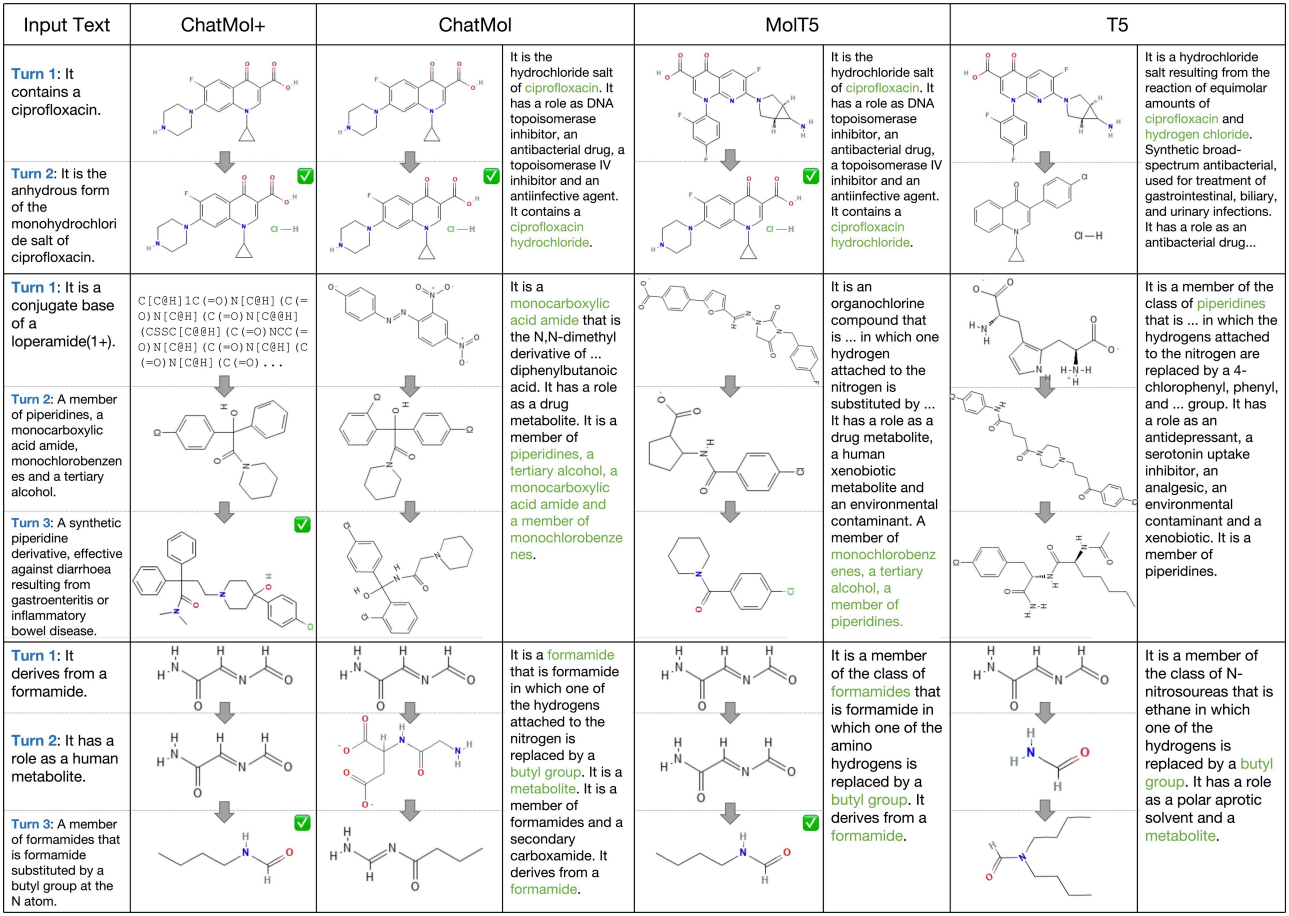
Case study for conversational molecular design. The correct molecules and properties are marked in tick mark and lighter text.

We provide several generation cases from the ChEBI-dia test set in Fig. 3. Both the intermediate results for the multi-turn input text descriptions and the generated property descriptions given the ground-truth molecules are shown.

As we can see, for the easy case (Line 1) that contains clear structural descriptions, all models provide high-quality molecule outputs from the first turn, and modify the molecule with the hydrochloride group according to the supplemented second turn information. For the comparably difficult case (Line 2) that provides more general and vague descriptions, baseline models fail to generate correct molecules and precise descriptions, while ChatMol+ eventually succeeds even when it makes a mistake in The first turn (which is not a valid molecule). ChatMol also grasps some key required substructures (e.g. piperidine, tertiary alcohol, monochlorobenzene) and generates a quite similar molecule, and meanwhile provides much more concise descriptions compared with baseline models. Nevertheless, the models trained in a targeted manner (MolT5 and our models) have overall better performances than T5 in many cases, as shown in Line 3.

**Figure 4.**
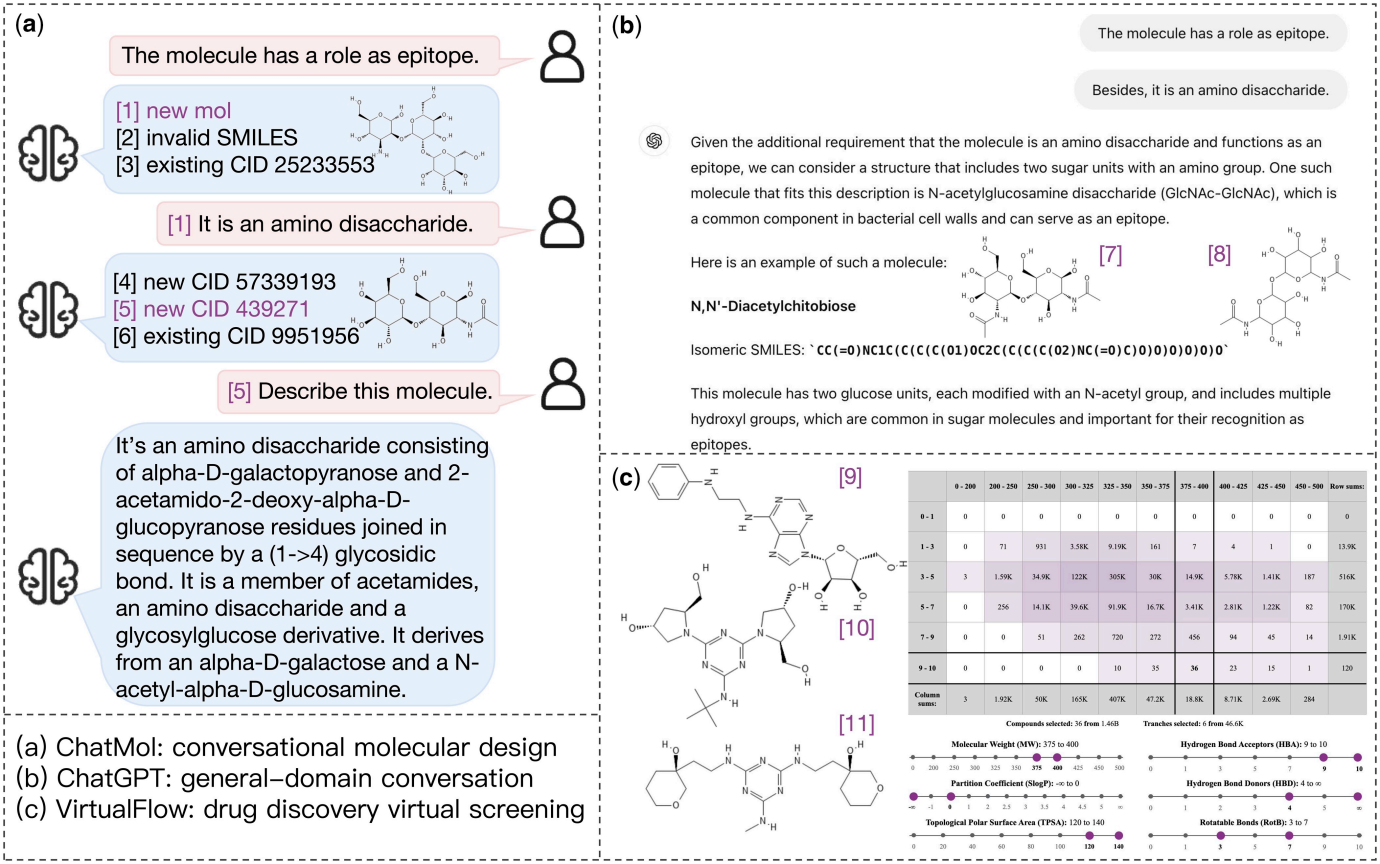
Practical comparison between our method and other systems.

We also compare ChatMol with other widely adopted molecular design systems in practical scenarios. As shown in Fig. 4, our method demonstrates generalization capability by generating molecules that were not present in the training data (e.g. Molecules 1, 4, and 5). In each turn of conversation, the model accurately understands the properties and successfully provides molecules that meet user requirements (Despras *et al.* 2012; Rossdam *et al.* 2024). In contrast, ChatGPT, representing general-domain LLMs, typically offers popular molecules from existing KBs and can sometimes produce satisfactory results based on user instructions. However, these models often lack the expertise to avoid hallucinations. For example, the name (Molecule 7) and the SMILES (Molecule 8) refer to two similar yet distinct molecules.

Traditional drug discovery platforms or software, such as VirtualFlow (Gorgulla *et al.* 2020), preprocess a vast number of molecules, allowing users to screen target molecules based on quantitative criteria such as molecular weight and hydrogen bond acceptors. When we attempt to retrieve molecules like *N*-acetyllactosamine (Molecule 5) using the method, we find that the number of hydrogen bond acceptors exceeds the upper limit. Consequently, we obtain 36 results, some of which (e.g. Molecule 9) have substructures that partially meet our requirements. However, this approach is not sufficiently convenient due to the limited options for screening properties.

Imagine the future where we can apply the technology of conversational molecular design for drug discovery, describing our command for structures and functions and obtaining possible molecules from the model outputs. Besides, readable descriptions can be generated when chemical researchers or learners input any unfamiliar molecules just as we do in the case study. Currently, the model and the data scales are both small, and the modifications and descriptions are also not so granular. It is possible to get stronger conversational molecular design systems that apply larger models with more fine-grained training data and have powerful knowledge memory to do more reliable predictions.

### Discussions

#### Methodological advantages

Our approach benefits from knowledge-enhanced training, which requires fewer steps and avoids issues such as compromised generalizability and instability, as noted in baseline analyses within experimental sections. Additionally, the integration of specialized tools, such as automatic identification of chemical substances and querying SMILES expressions, can reduce the model hallucination and improve the result accuracy. With the ongoing advancements in the capabilities of LLMs, the ability to leverage tools is becoming increasingly powerful and represents a significant trend. Moreover, our proposed interactive molecular design exemplifies the superiority of this task format. Interactive tasks are considerably more user-friendly, offering clear and intuitive advantages over traditional systems, as evident from specific case comparisons.

#### Methodological limitations

Though proved to be effective, our approach still has limitations, which can be broadly categorized into model- and data-related challenges. The former arises from our reliance on existing language models which, while proficient with natural language, do not inherently accommodate molecular representations. We employ natural language tokenizers, which have been enhanced with spatial-structure-related tasks, yet they are not ideally suited for encoding molecular data. Furthermore, for the sake of training and inference efficiency, we truncate the input and output text lengths, which leads to some application limitations even with the presence of large molecules in our dataset. Multistage training and accelerated inference can be considered to generalize our model to more scenarios.

The latter limitation pertains to the data-dependent nature of deep learning models. The quality of our model is tightly coupled with data quality. Due to a scarcity of manually annotated data (e.g. the chemical-natural language parallel data), we often resort to rule-based or model-assisted auto-annotation, which inevitably introduces noise. This phenomenon will help the model to improve its robustness to a certain extent. For example, when the user’s input contains typos or unclear instructions, our model can leave fuzzy space for small contradictions and generate results based on the overall encoding results of all inputs. However, when certain types of noise appear repeatedly in the training data, the model may also learn some incorrect knowledge, affecting overall performance.

#### Benchmark limitations

There is a paucity of widely accepted benchmarks in molecular understanding or designing tasks that acknowledge the need for flexible natural language interactions. Existing benchmarks such as MoleculeNet (Wu *et al.* 2018) focus on discrete properties prediction and do not emphasize interactions. Our attempts to establish a benchmark have faced challenges, including limited data volumes. With the increasing size of LLMs, T5-base level architectures may soon be deemed insufficient. Although our knowledge-enhancement techniques show promise, they could potentially be outpaced by brute force data and computing power. In fact, the large version of our baseline (MolT5) is reported to be much more powerful than the small and base versions, and this may indicate that various types of knowledge that we have discussed can be automatically learned and mastered once the computing and data resources are plenty.

Additionally, evaluation methods always remain inadequate for generative tasks. As we have observed, some descriptions are correct while not recorded in the ground truth texts, and conversely, a paragraph of descriptions might not only correspond to a single molecule. Therefore, it is hardly possible to thoroughly and reasonably evaluate model performance currently. While automated metrics provide some insight, the high cost and effort associated with manual evaluation limit our ability to discern fine-grained performance differences unless they are substantial. For text generation tasks, researchers have introduced automatic evaluation through LLMs (Wang *et al.* 2023b); however, for tasks related to molecular designing, the knowledge reserves of the LLMs themselves are still relatively limited and cannot be qualified for this task. Nevertheless, the current automated evaluation metrics, which assess the absolute similarity between predictions and ground truth answers, retain their validity. Particularly when all metrics exhibit consistent trends across multiple dimensions, these evaluation methods can scientifically and effectively demonstrate the efficacy of our approach.

#### Ethical and safety considerations

Deep learning systems operate as ‘black boxes’, with decision-making processes that are not fully transparent or interpretable. This is a significant concern in molecular design, which often intersects with critical chemical engineering or pharmaceutical processes requiring high safety standards. It is imperative that human experts play a central role in validating design outcomes and experimental results before they are applied in practice. At this stage, we view the AI system as a tool to expand human researchers’ capabilities and improve screening efficiency. This is also an important reason why we need to pay more attention to the convenience of human–computer interaction and introduce natural language.

Additionally, to prevent malicious individuals from using these systems to design harmful substances, it is essential that future AI systems with enhanced design capabilities incorporate techniques such as alignment to limit the range of acceptable commands. Furthermore, using AI to complete specialized tasks may also involve user permission and qualification controls to ensure that only authorized and qualified individuals can access and operate these systems.

#### Future research directions

Looking ahead, our research will focus on two main areas: Firstly, developing larger-scale, domain-specific models leveraging cutting-edge LLM technology, with targeted encoding and knowledge integration. Secondly, building AI systems that evolve through continuous human interaction, creating a virtuous cycle of data generation and model refinement in practical applications. Moreover, constructing such human–AI collaboration platforms is not only beneficial for molecular design but also holds potential for aiding in the full research process of high-throughput experiments, including automated execution, data analysis and mining, knowledge organization and publication, using larger-scale models with strong domain expertise in the future. However, apart from the technical improvement, it is also crucial for the communities to foster better integration and support, emphasizing user-friendly AI research and demonstrations, while encouraging chemical experts to embrace these systems with an open mind.

## Conclusion

In this paper, we propose conversational molecular design, an innovative interactive paradigm that utilizes natural language for describing and editing target molecules. We explore two specific tasks within this paradigm: Molecule understanding and molecule generation. To support these tasks, we have developed the dataset ChEBI-dia, tailored for conversational interactions. Furthermore, we present ChatMol, a knowledgeable generative model that effectively bridges chemical and natural language descriptions of molecules. By integrating molecular knowledge and facilitating the interaction between different language representations, ChatMol demonstrates enhanced effectiveness and efficiency, outperforming existing methods with a significantly reduced training cost. This approach heralds a promising new direction in AI-assisted molecular design, potentially transforming the field.

## Supplementary Material

btae534_Supplementary_Data

## Data Availability

Codes and data are provided in https://github.com/Ellenzzn/ChatMol/tree/main. PCdes, ChEBI-20, and S2orc are released under the CC BY-SA 4.0 license. All the datasets are used in a way consistent with their intended use. We observe the data samples and do not find any offensive content or identifiers in these datasets.
