# Supplementary material for: ChatMol: interactive molecular discovery with natural language
Source: Bioinformatics. 2024 Sep 2;40(9):btae534. doi: 10.1093/bioinformatics/btae534 (PMC11520398; doi:10.1093/bioinformatics/btae534)
Supplement: btae534_Supplementary_Data [file btae534_supplementary_data.pdf]

ChatMol: Interactive Molecular Discovery with Natural Language

Supplementary Data

0.1 Pre-training Corpus

For the pre-training period, we obtain the text corpus from S2orc (Lo et al., 2020), which contains over 12.7 million academic papers. We download SMILES strings from the PubChem database (Kim et al., 2016), which contains billions of substance records. The data between different tasks do not overlap.

For the SMILES corpus, we conduct three different tasks. (1) *SMILES MLM*: We sample SMILES strings for 300k molecules to conduct the basic MLM task. (2) *Molecular property prediction*: We collect 98k items of physical and chemical properties from wet-lab experiments of 31k molecules from PubChem. There are altogether 15 types of properties including Solubility, Color/Form, Boiling Point, Flash Point, Density, Vapor Density, Decomposition, Corrosivity, Melting Point, LogP, Vapor Pressure, Stability/Shelf Life, Odor, Taste, and pH. Notice that we also remain the SMILES-name pairs of these molecules to conduct mapping correlation learning. (3) *Spatial structure learning*: We retain the other 300k molecules from PubChem. 15% of the atoms in each molecule are randomly marked and required to recognize their connected atoms, aromaticity and ring formation information.

For the text corpus, we conduct two types of tasks. (1) *Text MLM*: We select 400k literature abstracts in the chemistry and biomedicine field for the basic MLM task. (2) *Mapping correlation learning*: There are two methods for automatically obtaining parallel data between text and molecules. The first one is to directly match the name-SMILES pairs, and we sample 200k molecules for it. The second one is to roughly annotate the corresponding entity SMILES in the corpus. Specifically, we adopt SciSpacy (Neumann et al., 2019) to find out the chemical entities, which are then matched with the list of substances and synonyms from PubChem. In this way, we get over 2.4 million mentions of 4.7k most frequently-appeared molecules linked with their SMILES strings in 760k literature abstracts.

For the dual augmentation, we adopt the 14.7k molecules from ChEBI-20 train set that are not contained in ChEBI-dia as the augmented pool, while do not use their text descriptions to avoid introducing extra information.

0.2 Intermediate turn analysis

We provide the intermediate prediction results of the multi-turn molecule generation task in Table 1. Since the intermediate answers are annotated automatically, their accuracy cannot be guaranteed and should be used as a reference only. From the results we can conclude that: (1) We use the MolT5 initial checkpoint to annotate intermediate answers, and naturally the fine-tuned MolT5 generates similar molecules and achieves a quite satisfying performance. Though ChatMol gets a lower score, the plugin version ChatMol+ does even better than MolT5, showing the great assistance that the chemical-natural language mapping tool (chemical entity recognition toolkits and SMILES KBs) can provide; (2) Early turns have high accuracy, indicating that it is easy for the models to find molecules that meet the requirements for the general descriptions, while it is more difficult to complete the modification for the supplementary fine structure descriptions.

0.3 Training Setting Supplementary

We initialize ChatMol with the pre-trained T5-base (with the hidden size of 768). Other hyperparameter details can be found in the original paper (Raffel et al., 2020). Molecule understanding and generation correspond to two different checkpoints. We implement our method in the PyTorch (Paszke et al., 2019) framework, and adopt the Huggingface Transformers (Wolf et al., 2019). We take the

Model	Turn	EM	BL	RDK
ChatMol+	1	**0.402**	**0.716**	**0.672**
	2	**0.211**	**0.691**	**0.592**
	3	**0.190**	**0.716**	**0.614**
	4	0.125	0.716	0.626
ChatMol	1	0.393	0.568	0.638
	2	0.192	0.532	0.573
	3	0.092	0.522	0.578
	4	0.176	0.587	**0.636**
MolT5	1	0.384	0.689	0.635
	2	0.194	0.650	0.559
	3	0.135	0.657	0.536
	4	**0.206**	**0.717**	0.598
T5	1	0.367	0.676	0.630
	2	0.162	0.630	0.540
	3	0.130	0.669	0.539
	4	0.118	0.678	0.503

Table 1: Intermediate results for molecule generation.

AdamW optimizer (Loshchilov and Hutter, 2019) which is suitable for most of the PTMs in the T5 backbone.

For the multi-task pre-training, we add different task prefixes (e.g. "Predict Solubility:") to the input, and randomly mix up the training data for MLM, SMILES-text parallel generation, property and spatial information prediction. In this period, we set the learning rate as $5e-4$ and batch size as 256.

For the downstream molecule understanding fine-tuning, we set the maximum epoch number as 50, and the early stop epoch number as 5. The learning rate and batch size is searched in $\{1e-3, 5e-4, 2e-4\}$ and $\{8, 16, 32\}$, and eventually decided as $5e-4$ and 16. Notice that our hyper-parameter settings are different from the MolT5 paper (Edwards et al., 2022), therefore the results are higher than original reported. For molecule generation, the learning rate and batch size is searched in $\{1e-3, 5e-4, 1e-4, 5e-5\}$ and $\{16, 32, 64\}$, and eventually decided as $5e-4$ and 32.

We train our models on a NVIDIA A100 SXM4 40 GB GPU. Evaluation metrics such as BLEU and ROUGE are achieved with the assistance of NLTK toolkit (Loper and Bird, 2002).

0.4 Licenses and Data Usage Policy

PCdes, ChEBI-20 and S2orc are released under the CC BY-SA 4.0 license. MoleculeNet are released under the MIT license. All the datasets are used in a way consistent with their intended use. We observe the data samples and do not find any offensive content or identifiers in these datasets.

References

Carl Edwards, Tuan Lai, Kevin Ros, Garrett Honke, and Heng Ji. 2022. Translation between molecules and natural language. *arXiv preprint*.

Sunghwan Kim, Paul A Thiessen, Evan E Bolton, Jie Chen, Gang Fu, Asta Gindulyte, Lianyi Han, Jane He, Siqian He, Benjamin A Shoemaker, et al. 2016. Pubchem substance and compound databases. *Nucleic acids research*, 44(D1):D1202–D1213.

Kyle Lo, Lucy Lu Wang, Mark Neumann, Rodney Kinney, and Daniel S Weld. 2020. S2orc: The semantic scholar open research corpus. In *Proceedings of the 58th ACL*, pages 4969–4983.

Edward Loper and Steven Bird. 2002. Nltk: The natural language toolkit. In *Proceedings of the ACL-02 Workshop on Effective Tools and Methodologies for Teaching Natural Language Processing and Computational Linguistics*, pages 63–70.

Ilya Loshchilov and Frank Hutter. 2019. Decoupled weight decay regularization. In *International Conference on Learning Representations*.

Mark Neumann, Daniel King, Iz Beltagy, and Waleed Ammar. 2019. Scispacy: Fast and robust models for biomedical natural language processing. In *Proceedings of the 18th BioNLP Workshop and Shared Task*, pages 319–327.

Adam Paszke, Sam Gross, Francisco Massa, Adam Lerer, James Bradbury, Gregory Chanan, Trevor Killeen, Zeming Lin, Natalia Gimelshein, Luca Antiga, et al. 2019. Pytorch: An imperative style, high-performance deep learning library. *Advances in NeurIPS*, 32.

Colin Raffel, Noam Shazeer, Adam Roberts, Katherine Lee, Sharan Narang, Michael Matena, Yanqi Zhou, Wei Li, Peter J Liu, et al. 2020. Exploring the limits of transfer learning with a unified text-to-text transformer. *Journal of Machine Learning Research*, 21(140):1–67.

Thomas Wolf, Lysandre Debut, Victor Sanh, Julien Chaumond, Clement Delangue, Anthony Moi, Pierric Cistac, Tim Rault, Rémi Louf, Morgan Funtowicz, et al. 2019. Huggingface's transformers: State-of-the-art natural language processing. *arXiv preprint*.
